# Editorial: Psychotropic overprescribing to youth: scope of the problem, causes, and possible solutions

**DOI:** 10.3389/fpsyt.2024.1418600

**Published:** 2024-05-06

**Authors:** Jennifer L. McLaren, Julie M. Zito, Jörg M. Fegert, Erin R. Barnett

**Affiliations:** ^1^ Department of Psychiatry, Dartmouth Health, Lebanon, NH, United States; ^2^ Department of Pediatrics, Dartmouth Health Children's, Lebanon, NH, United States; ^3^ The Dartmouth Institute for Health Policy & Clinical Practice, Geisel School of Medicine at Dartmouth, Hanover, NH, United States; ^4^ Department of Practice, Sciences and Health Outcomes Research, University of Maryland, Baltimore, Baltimore, MD, United States; ^5^ Department of Psychiatry, School of Medicine, University of Maryland, Baltimore, Baltimore, MD, United States; ^6^ Department of Child and Adolescent Psychiatry and Psychotherapy, University of Ulm, Ulm, Germany

**Keywords:** psychotropic, youth, overprescribing, polypharmacy, antipsychotic, foster care

For more than 20 years, psychotropic medication trends have been steadily increasing for youth (1–9). The United States leads the world in high prevalence rates of use. In Europe and Australia, the trend of rising psychotropic medication rates exists, albeit at a moderate pace. There is notable variation in psychotropic prescription rates across different countries, influenced by historical attitudes, disparities in healthcare access, and varying guidelines (10–17). The accessibility and acceptance of a biological psychiatry treatment model have contributed to poorly evidenced treatments becoming common for pediatric mental health services. Most concerning is the fact that the number of youth receiving antipsychotics has increased by 50%-200% over the past 20 years, depending on the cohort (1, 3–5). Further, most of the antipsychotic use in youth targets non-psychotic conditions and have not received approved or licensed labeling, thus, is ‘off-label’ or unapproved. Polypharmacy, i.e. combining classes of psychotropics, despite the lack of robust evidence that benefits outweigh risks, has also sharply increased (1, 4–9). As with antipsychotics for behavioral use, 3-class polypharmacy lacks evidence that benefits outweigh risks (9).

Unfortunately, parents are seldom aware of the risks (e.g. weight gain, metabolic changes, diabetes, sedation, tremor, somnolence, and restlessness) associated with these medications and, at times, are not fully engaged in the decisions to use them (18, 19). The effects of polypharmacy are largely unstudied, and there are significant concerns about drug interactions (20–22).

The routes to “too many, too much, too young” psychotropic medication use in youth are numerous, yet the routes to reducing them are just being forged. Some government agencies, health organizations, and media outlets have recommended reducing the use of high-risk medications and medication combinations (23–26). In addition, some preliminary research suggests that clinicians may be eager to remove or stop medications when appropriate but lack guidance from the field on how to do so (26). Clinicians also report perceived parental concerns about reducing medications, although some research suggests that parents are interested in deprescribing (18, 27).

This Research Topic contains five papers from 22 authors from Europe and the United States. There are original research articles, a brief research report, a perspective, and a commentary. It highlights research on the overuse of and potential reduction in psychotropic medications for youth and contains commentaries on diagnostic conceptualization.

Two papers describe population-based pharmacoepidemiology studies in Germany and the USA. Dörks et al. analyzed outpatient claims data from Germany between 2011 to 2020 and found an increase in antipsychotic use in youth. Child psychiatrists increasingly prescribed antipsychotics during the study period. Risperidone was most often prescribed to males with attention-deficit hyperactivity disorder and quetiapine was most commonly prescribed to females with depression. The increased antipsychotic utilization among German youth for off label indications is concerning and calls for an examination of potential factors, such as limited access to psychosocial interventions and the need for oversight and prescribing protocols. Cosme et al. examined psychotropic prescribing patterns in youth aged 2 to 19 years in foster care in Nevada, USA. They analyzed data from an institution’s electronic medical records from July 2019 to June 2022 and reviewed 569 distinct psychotropic treatment episodes. They found prevalent use of psychotropic polypharmacy, non-stimulant ADHD medications, atypical antipsychotics, and antidepressants. Further research is needed to understand the reasons behind these prescribing practices and the implications for youth.

Two papers share their perspectives on ADHD. Banaschewski et al. challenge the notion of ADHD as exclusively a natural entity and note that societal and environmental factors need to be considered. They stress that recognizing ADHD as a social construct is crucial for diagnosis and treatment decisions, emphasizing the need for a person-centered approach within a context-dependent model. Dekkers‘ commentary agreed with the suggestions from Banaschewski et al., emphasizing a paradigm shift towards viewing ADHD as a social construct, aiming to address overmedication and stigma. Dekkers argues that decontextualizing ADHD from solely biological causes may reduce prognostic pessimism, stigma, and overreliance on psychotropics, and thus offer more holistic support for youth. These proposed approaches would move away from ‘medicalized’ care to a wider sociological orientation steeped in the socioeconomic and educational context of youth’s and families’ lives.

Finally, Monson et al. describe and evaluate The Utah Psychotropic Oversight Program (UPOP) for prescribers caring for foster care youth in the USA. The study analyzed 8,523 youth over 4 years. Fostered youth receiving antipsychotics through UPOP tended to be older males with disruptive behavior disorders and high rates of polypharmacy. With the oversight program prescription rates decreased over time without raising the need for higher levels of care. The study suggests that oversight programs like UPOP can influence prescribing practices.

This Research Topic highlights concerns regarding psychotropic use among youth, particularly some of the most vulnerable youth, those involved in the foster care system. Dörks et al., Monson et al. and, Cosme et al. all emphasize the prevalence of antipsychotics and/or polypharmacy and the need to understand prescribing patterns and their implications. Monson et al. underscores the importance of oversight programs and their potential influence on prescribing practices. It is important to note, that in many states and countries, government and payor initiatives have already initiated oversight programs and experts have developed guidance around deprescribing psychotropic medications in youth (28, 29). Banaschewski et al. and Dekkers challenge the notion of ADHD as purely biological and advocate for a broader and more nuanced understanding that considers societal and environmental factors. Ultimately, these studies collectively emphasize the need for a more integrated youth centered approach to balance psychopharmacologic and psychotherapeutic treatment, particularly for those in out of home placement by youth welfare services (e.g. foster care, looked after children), while also acknowledging the influence of broader social and environmental contexts on diagnosis and treatment decisions.

## Author contributions

JM: Writing – original draft, Writing – review & editing. JZ: Writing – original draft, Writing – review & editing. JF: Writing – original draft, Writing – review & editing. EB: Writing – original draft, Writing – review & editing.
